# Synthesis and Characterization of a Ru(II) Complex with Functionalized Phenanthroline Ligands Having Single-Double Linked Anthracenyl and 1-Methoxy-1-buten-3-yne Moieties

**DOI:** 10.3390/molecules15117570

**Published:** 2010-10-27

**Authors:** Adewale O. Adeloye, Peter A. Ajibade

**Affiliations:** Department of Chemistry, Faculty of Science and Agriculture, University of Fort Hare, Private Bag X1314, Alice 5700, South Africa

**Keywords:** Ru(II) complex, phenanthroline, extended π-bond conjugation, spectroscopy, molar extinction coefficient

## Abstract

Two series of bidentate polypyridine ligands, made of phenanthroline chelating subunits having substituted mono-and di-anthracenyl groups, and 1-methoxy-1-buten-3-yne at the 4 and 7-positions with the corresponding heteroleptic Ru(II) complex have been synthesized and characterized. The complex is formulated as [(Ru(L_1_)(L_2_)(NCS)_2_)], (where L_1_ = 4-(9-dianthracenyl-10-(2,3-dimethylacrylic acid)-7-(9-anthracenyl-10-(2,3-dimethylacrylic acid)-1,10-phenanthroline and L_2_ = 4,7-bis(1-methoxy-1-buten-3-yne)-1,10-phenanthroline). The Ru(II) complex shows characteristic broad and intense metal-to-ligand charge transfer (MLCT) bands absorption and appreciable photoluminescence spanning the visible region. The ligands and complex were characterized by FT-IR, ^1^H, ^13^C NMR spectroscopy, UV-Vis, photoluminescence and elemental analysis (see in supplementary materials). The anchoring groups in both ligands have allowed an extended delocalization of acceptor orbital of the metal-to-ligand charge-transfer (MLCT) excited state.

## 1. Introduction

Coordination chemistry is about the influence of the ligands on the chemical properties of metal ions. These include stabilization of different oxidation states and modulation of the electrophilic and nucleophilic properties of the metals. However, in spite of the influence of the ligands on the metal ions, it is striking how often certain distinctive properties of a given metallic element persists through drastic ligand changes [1]. Tuning the optical properties of transition metal complexes by ligand tailoring has been a fascinating research field which has generated highly coloured pigments, very efficient triplet energy and electron transfer reactions, long-lived excited states, charge separated species, and singlet oxygen producers [2]. Along these lines, polyaromatic modified bipyridines, and phenanthrolines have extensively been studied and interesting features connected to lifetime enhancement from the excited state manifold has been reported. Ruthenium(II) polypyridyl complexes have attracted attention in recent years due to their well-defined spectroscopic, photophysical, photochemical and electrochemical properties [3]. These properties are of particular use in the construction of supramolecular systems [4] and in the development of photochemically driven molecular devices [5]. The ability to tune the excited state properties of these complexes is central to their potential for practical applications. An important issue concerns how best to design the spacer that permits a controlled transfer of electron or energy along the molecular axis. In principle, this issue can be resolved by careful manipulation of the energetic of the end-capping metal centres and the connecting spacer. In general, the design principle combines the most unsaturated form of organic linear spacer with the most stable redox- or photo-active terminals [4,5,6,7,8,9,10,11,12,13,14,15,16,17,18,19,20]. In this paper, we report the synthesis of a heteroleptic ruthenium(II) phenanthroline complex. Here, we envisioned that the introduction of anthracene derivative and/or a 1,3-enyne moiety as substituents of the phenanthroline thereby extending the π-conjugation length will show an enhanced photophysical properties of the ruthenium(II) complex because the antenna units have the role of absorbing the incident light and transferring the electronic energy of the sensitizer fragment. The strategy adopted however is in line with the continuation of the efforts geared towards the synthesis of ruthenium(II) complex sensitizers that may be used in energy and electron transfer processes, dendrimers for light harvesting and light-powered molecular machine.

## 2. Results and Discussion

### 2.1. Chemistry

**Scheme 1:**
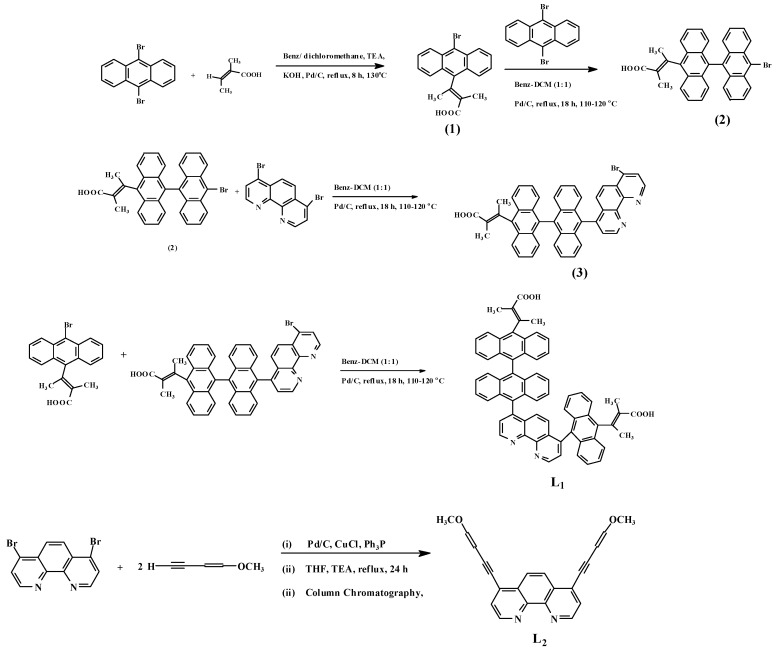
Synthesis of **L**_1_ and **L**_2_.

**Scheme 2:**
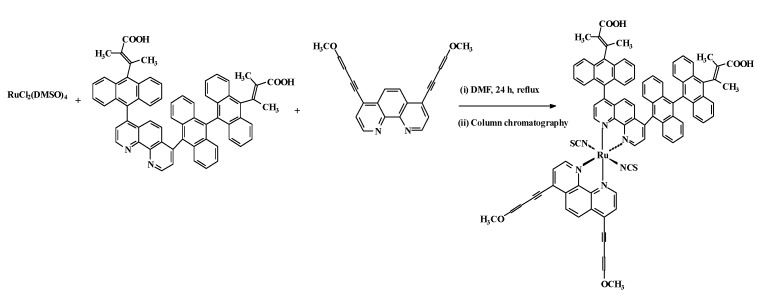
Synthesis of complex Ru**L**_1_**L**_2_(NCS)_2_.

Scheme 1 and Scheme 2 show the stepwise synthetic pathways and outline the chemistry of the present study. 9,10-Dibromoanthracene, 2,3-dimethylacrylic acid and 4,7-dibromo-1,10-phenanthroline are the starting materials for **1**, **2**, **3** and **L_1_** following with slight modifications the well established procedures reported in the literature [21,22,23,24]. 9-Bromo-10-(2,3-dimethylacrylic acid) (**1**) was obtained when 9,10-dibromoanthracene and 2,3-dimethylacrylic acid were refluxed in a benzene/dichloromethane mixture under basic conditions using triethylamine, potassium hydroxide and palladium carbide. Further treatment of **1** with 9,10-dibromoanthracene using the same reaction conditions afforded **2** after recrystallization from diethyl ether. Reaction of **2** with 4,7-dibromo-1,10-phenanthroline in benzene/ dichloromethane under palladium catalyzed cross reaction conditions gave **3**. Subsequently, a stoichiometric addition of **1** and **3** under the same reaction conditions as reported previously afford the desired **L_1_** as a yellowish-green solid. **L_2_** was synthesized following the methodology reported by Venkataraman *et al*. [25]. It is to be noted that all anthracenyl intermediate products showed good solubility properties in common organic solvents, unlike the 9,10-dibromoanthracene starting reagent. The ruthenium complex of the ligands was prepared as reported in the literature [26,27]. [RuCl_2_(dmso)_4_] was dissolved in dimethylformamide and sequentially, **L_1_**, **L_2_** and ammonium thiocyanate were added. The mixture was refluxed overnight under argon, and finally purified by column chromatography in Sephadex LH-20 using methanol-ether as eluant.

### 2.2. Infrared spectra

**Figure 1:**
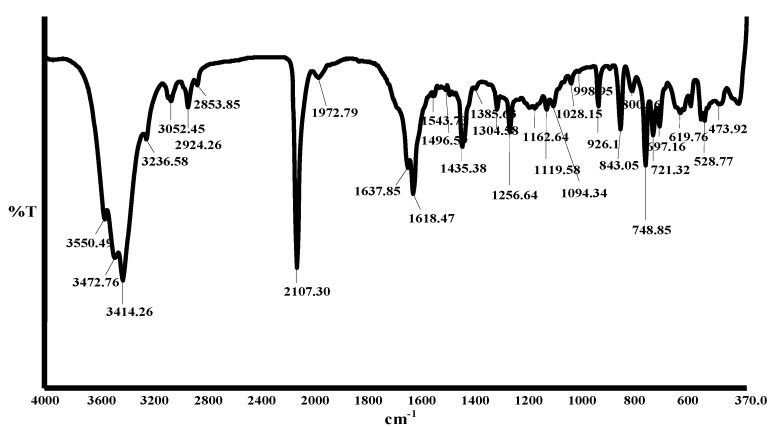
FT-IR spectra of [RuL_1_L_2_(NCS)_2_] in KBr.

The infrared spectra of the starting material, the ligands and the [RuL_1_L_2_(NCS)_2_] complex were studied and bands assigned after careful comparison. The complex showed a broad band in the 3550-3400 cm^-1^ region which is due to the O-H vibrational stretch characteristic of α,β-unsaturated carboxylic acids and the possibility of intramolecular hydrogen bonding between the free COOH groups. This band is shifted slightly to lower frequency and of reduced intensity in L_1_ (Figure 1).

A band at 3,052 cm^-1^ was assigned to the C-H stretching vibrations of a substituted alkene found in L_2_. Common to the ligands and the complex were two stretching vibrational bands at 2,925 and 2,853 cm^-1^, which were assigned to the methyl groups in the molecules. The complex shows a broad absorption frequency at 2,107 cm^-1^ for stretch vibrational modes due to the N-coordinated ν(NCS). Because of the stronger bonding, the absorption of the triple bond vibration occurs in the region between 2,300-2,000 cm^-1^, far removed from that of single and double bonds. As a result of the small change in the dipole moment during vibration, however, monosubstituted acetylenes show only a weak band, unlike in **L_2_** that was completely missing and/or overlap with the C=N absorption band of the thiocyanate group in the complex due to di-substitution [28]. The bands at 1,972, 1,637 and 1,304 cm^-1^ were assigned to the ν(C=O) and ν(C-O) stretching of carboxylic acid group, respectively. The bands at 1,618, 1,543 and 1,496 cm^-1^ are due to ring stretching modes of the ligands. The bands at 1,435, 1,385 and 1,028 cm^-1^ were assigned to the (C=C) alkene moiety in **L_2_**, the (-COO^-^) of the carboxylic acid group and the (C-O) of the ethereal group in **L_2_**, respectively. A comparison of the infrared spectra of **L_1_** and 9,10-dibromoanthracene showed that a strong vibrational band in the former was conspicuously absent in the latter, confirming the loss of C-Br bond and the formation of C-C bond linkages of the polyanthracenyl group. Furthermore, the C-C bond linkage between anthracene and phenanthroline was affirmed by the absorption frequency at 853 cm^-1^ which was shifted to lower frequency by 10 cm^-1^ in the complex. Peaks in the region 748 and 697 cm^-1^ demonstrate the existence of four neighbouring hydrogen atoms as observed in the *para*-substitution pattern of the fused anthracene rings in **L_1_** and the [RuL_1_L_2_(NCS)_2_] complex. All vibrational peaks in the region are found relatively weak and broad in the complex, which may be ascribed to the loss of crystallinity and the broad distribution of the anthracene chain length [29]. The weak absorption frequencies at 528 and 473 cm^-1^, respectively, show the coordination of nitrogen atoms of the ancillary ligands to ruthenium central metal atom [30].

### 2.3. NMR spectroscopy

**Figure 2:**
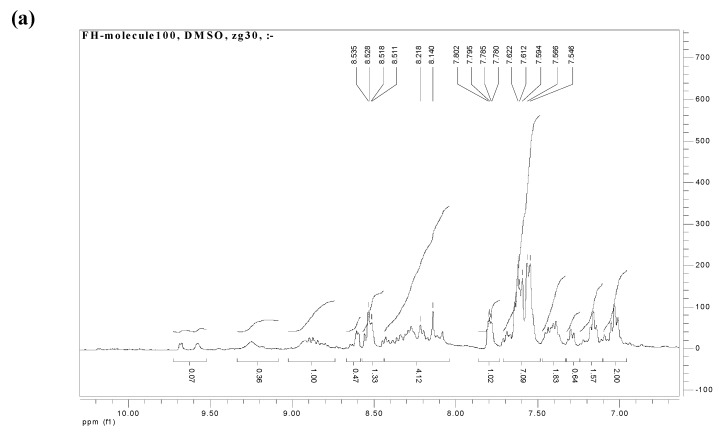

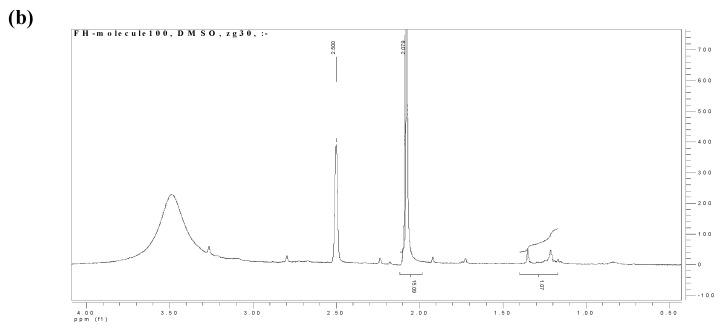
^1^H-NMR spectra of complex [RuL_1_L_2_(NCS)_2_]. (a) Aromatic region ^1^H-NMR spectrum of [RuL_1_L_2_(NCS)_2_] complex; (b) aliphatic region ^1^H-NMR spectrum of [RuL_1_L_2_(NCS)_2_] complex.

The molecular structure of the [RuL_1_L_2_(NCS)_2_] complex was confirmed by ^1^H-NMR (Figure 2). The ^1^H-NMR multiplet peaks in the aromatic region [Figure 2(a)] showed a total number of hydrogens integrated for twenty-one protons due to chemical equivalency. Compared to the two ligands L_1_ and L_2_ respectively, the protons in the complex shifted upfield by (ca δ 0.63, 0.48 ppm). The peaks at δ 8.53 (d, *J* = 2.8 Hz, 1H), δ 8.52 (d, *J* = 2.8 Hz, 1H), and δ 7.62 (d, *J* = 4.0 Hz, 1H) were unambiguously assigned to H-2,9, H-2',9'; H- 5,6; 5',6' and 3,8, H-3',8', of the two ancillary phenanthroline ligands respectively. The different substitution patterns in L_1_ of the complex gave peaks at δ 8.17 (m, 4H) and δ 7.79 (dd, *J* = 2.8, 6.8 Hz, 8H) for the anthracene protons; while the two signals at δ 7.58 (d, *J* = 11.2 Hz, 2H) and δ 7.56 (d, *J* = 8.0 Hz, 2H) were assigned to the *trans* olefinic protons of the 1,3-enyne moiety in L_2_. In the upfield aliphatic region [Figure 2(b)] the eighteen methyl protons in the complex were integrated as a singlet peak for fifteen protons at δ 2.08 ppm. This could be explained in terms of the equivalency of the methyl protons of the -OCH_3_ group in L_2,_ unlike in L_1_ where the methyl groups of the 2,3-dimethyacrylic acid were orientated in different environments on the phenanthroline ring.

### 2.4. Electronic and emission spectra

**Figure 3:**
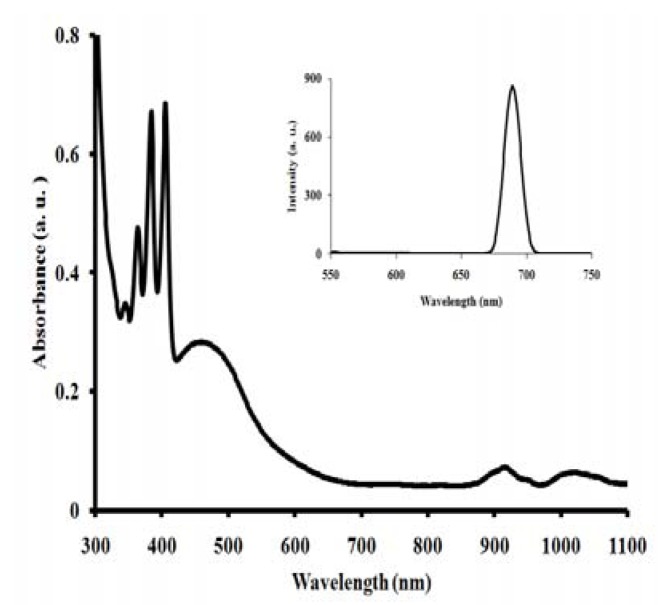
UV**-**Vis absorption and luminescence (inset) spectra of the [RuL_1_L_2_(NCS)_2_] complex at a concentration of 0.001 g/dm^3^ in dimethylformamide.

The UV-Vis absorbance and emission spectra of the [RuL_1_L_2_(NCS)_2_] complex in dimethylformamide are presented in Figure 3. In the UV-region, the [RuL_1_L_2_(NCS)_2_] complex displays four distinct vibronic peaks for the intra ligand (π→π*) charge transfer transitions characteristics of anthracene derivatives at 343, 363, 384 and 405 nm [34]. The [RuL_1_L_2_(NCS)_2_] complex shows broad and intense absorption band between 419 and 577 nm with wavelength maximum at 461 nm (ε = 41,400 M^-1^cm^-1^). This absorption is ascribed to the spin-allowed metal-to-ligand charge transfer transitions (MLCT) [34]. The molar absorptivity coefficient of the complex was enhanced in the visible region due to the extension of the π-bond conjugation created by the attachment of polyanthracenyl groups on the one hand and the 1,3-enyne molecular unit on the other hand as found in the two phenanthroline ligands [18,20]. In the long wavelength tail of the absorption spectrum, small but significantly distinct shoulders at 915 nm (ε = 10,570), and 1,008 nm (ε = 9,080) were observed. These absorption features are thought to correspond to the lowest ^3^MLCT excited states [1]. Groups which extend the delocalization of the π systems of polycyclic arenes cause further bathochromic shifts, but the extent of these shifts vary with the positions of substitution [35].

Upon excitation into the ^1^LC and ^1^MLCT bands, (λ_exc_ = 550 nm), the [RuL_1_L_2_(NCS)_2_] complex displays appreciable luminescence at room temperature (λ_em_ = 690 nm) (Figure 3 inset). It is well known that conjugated functional organic molecules are useful for the study of electron transport at the molecular scale and that the use of fused-ring systems is a powerful and practical approach [36,37]. The luminescent properties of a complex as well as its ability to play the role of excited state reactant or product are related to the energy ordering of its low energy excited state and, particularly, to the orbital nature of its lowest excited state. With the choice of ligands, it is well thought that the energy positions of MC, MLCT, and LC excited states of RuL_1_L_2_(NCS)_2_] complexes depend on the ligand field strength [1]. The B3LYP/6-31G theoretical calculations showed that the electronic structures of anthracene derivatives are perturbed by the side substitutes on the anthracene block, and the slight variation of the electronic structures results in the enhanced electron accepting ability and the decrease of the HOMO-LUMO energy gap, which is the origin of the shifting of emission wavelength to the blue-green region [38].

## 3. Experimental

### 3.1. Materials and general physical measurements

All commercial reagents used were analytically pure and used without further purification. 4,7-Dibromo-1,10-phenanthroline was synthesized as described in the literature [21]. 4,7-bis(1-Methoxy-1-buten-3-yne)-1,10-phenanthroline, 4-(dianthracenyl-2,3-dimethylacrylic acid)-7-(anthracenyl-2,3-dimethylacrylic acid)-1,10-phenanthroline and the [RuL_1_L_2_(NCS)_2_] complex were synthesized with modifications to the reported procedure (Scheme 1 and Scheme 2). All thin layer chromatography (TLC) analyses were done with aluminium sheets precoated with normal phase silica gel 60 F_254_ (Merck, 0.20 mm thickness) unless otherwise stated. The TLC plates were developed using any of the following solvent systems: solvent system A: dichloromethane-methanol (9:1); solvent system B: dichloromethane-methanol (7:3); solvent system C: dichloromethane-benzene (3:7); solvent system D: chloroform-methanol (1:1), and solvent system E: *n*-hexane-dichloromethane (1:1). Gel filtration was performed using Sephadex LH-20 previously swollen in specified solvent (s) prior to loading of extract onto the column (3.5 cm × 8.5 cm).

Melting points were determined using a Gallenkamp electrothermal melting point apparatus. Microanalyses (C, H, N, and S) were carried out with a Fisons elemental analyzer and infrared spectra were obtained with KBr discs or Nujol on a Perkin Elmer System 2000 FT-IR Spectrophotometer. UV-Vis and fluorescence spectra were recorded in a 1 cm path length quartz cell on a Perkin Elmer Lambda 35 spectrophotometer and Perkin Elmer Lambda 45 spectrofluorimeter, respectively. ^1^H- and ^13^C-Nuclear Magnetic Resonance (NMR) spectra were run on a Bruker EMX 400 MHz spectrometer for ^1^H and 100 MHz for ^13^C. The chemical shift values were reported in parts per million (ppm) relative to (TMS) as internal standard. Chemical shifts were also reported with respect to DMSO d_6_ at δ_c_ 40.98 and DMSO d_6_ at δ_H_ 2.50 or CDCl_3_ at δ_c_ 77.30 and δ_H_ 7.24 for synthesized ligands and complex.

### 3.2. Synthesis of ligands

#### *3.2.1.* 4-(Dianthracenyl-2,3-dimethylacrylic acid)-7-(anthracenyl-2,3-dimethylacrylic acid)-1,10-phenanthroline *(**L**_1_)*

In a 250 mL round bottom flask, 9,10-dibromoanthracene (0.37 g, 1.10 mmol) and 2,3-dimethyl-acrylic acid (0.20 g, 1.10 mmol) were dissolved in benzene/dichloromethane (75 mL, v/v, 1:1) followed by addition of of triethylamine (1.0 mL), KOH (0.06 g, 1.10 mmol) and palladium carbide (0.05 g). After 8 h reflux, the mixture was filtered and concentrated to dryness *in vacuo*. Degassed water (30 mL) was added and the yellow organic solid precipitated at the bottom of the flask and was extracted exhaustively with chloroform. The chloroform extract was concentrated *in vacuo*, and recrystallized from a 50% diethyl ether-ethanol mixture to afford a brilliant yellow solid **1**. In another reaction, an equivalent molar ratio of the yellow solid product **1** and 9,10-dibromoanthracene were dissolved in benzene/dichloromethane (100 mL, v/v, 1:1) and refluxed at 110-120 ºC for 18 h under palladium/carbide cross-catalyzed reaction conditions using a modified reaction method as reported by Yamamoto and co-workers [22]. A light-green solid product **2** was obtained as a bromodianthracenyl-2,3-dimethylacrylic acid derivative after recrystallization from diethyl ether. Following a one reaction synthetic process; an equivalent molar ratio of 9-bromodianthracenyl-2,3-dimethylacrylic acid (0.78 g, 1.48 mmol), 4,7-dibromo-1,10-phenanthroline (0.49 g, 1.48 mmol) and 9-bromoanthracenyl-2,3-dimethylacrylic acid in benzene/dichloromethane (v/v, 1:1) were reacted under reaction conditions similar to those described above to afford a yellow-green solid product [(4-dianthracenyl-2,3-dimethylacrylic acid)-(7-anthracenyl-2,3-dimethylacrylic acid)-1,10-phenanthroline] (**3**). Yield 55.65%, m.p. 203-205 ºC; IR (KBr): 3,381, 3,027, 2,925, 1,929, 1,621, 1,587, 1,502, 1,422, 1,346, 1,304, 1,255, 1,217, 1,137, 1,091, 1,026, 925, 853, 778, 746, 675, 623, 578; ^1^H-NMR (DMSO-d_6_): δ 9.10 (d, *J* = 4.0 Hz, H-2, 9), 7.98 (d, H-3, 8), 8.36 (s, H-5, 6), 8.48 (dd, *J* = 8.0 Hz, 4H), 7.77 (m, 4H), 2.08 (s, CH_3_). ^13^C-NMR (DMSO-d_6_) δ 150.38, 150.28, 146.30, 146.21, 138.79, 136.21, 136.13, 128.81, 128.72, 126.68, 126.59, 123.27, 123.19, 14.74, 12.14; Elemental analysis: Calc: C, 84.93; H, 4.90; N, 3.10. Found: C, 84.66; H, 5.15; N, 3.55. (Mol. Formula, C_64_H_44_N_2_O_4_).

#### *3.3.2.* 4,7-bis(1-Methoxy-1-but-3-yne)-1,10-phenanthroline *(**2**)*

The synthesis of **L_2_** followed a modified synthetic method as reported by Venkataraman and co-workers [25]. In an argon-filled standard Schlenk tube, Pd/C (0.338 mmol, 0.8%), copper chloride (0.084 g, 0.844 mmol, 2.0%) and triphenylphosphine (1.107 g, 4.22 mmol, 10.0%) were added to a thick-walled glass tube and tetrahydrofuran (THF, 50 mL) was added to form a green solution. The mixture was stirred vigorously for 10 min prior to addition of triethylamine (7.50 mL), 4,7-dibromo-1,10-phenanthroline (3.5 mmol) and 1-methoxy-1-buten-3-yne (7.0 mmol). The mixture was refluxed under argon at 100-110 ºC for 24 h. After reaction was complete, the crude product was filtered twice and the red filtrate concentrated *in vacuo* to afford red oil. The red oil solidified upon cooling to room temperature and was redissolved in dichloromethane, before adsorption onto a silica gel for column chromatography. The solid was eluted using *n*-hexane-dichloromethane (1:1, v/v) mixture to afford **L_2_** as a reddish-brown semi-solid. This was used for complexation without any further purification. Yield 56.8%; R_f_ = 0.67 (*n*-hexane-dichloromethane, 1:1, v/v); IR (KBr): 3,478, 3,416, 3,063, 2,931, 2,039, 1,638, 1,619, 1,589, 1,504, 1,421, 1,345, 1,219, 1,091, 852, 738, 696, 624, 541, 496; ^1^H-NMR (CDCl_3_): δ 9.16 (d, *J* = 3.6 Hz, 2H, H-2, 9), 8.19 (d, *J* = 8.0 Hz, 2H, H-3, 8), 7.72 (s, 2H, H-5, 6), 7.58 (d, *J* = 3.6 Hz, 2H, H-1^a^), 7.30 (d, *J* = 3.6 Hz, 2H, H-1^b^); ^13^C-NMR (CDCl_3_): δ 150.67, 146.61, 137.56, 137.45, 136.36, 134.20, 132.51, 132.41, 132.37, 129.14, 128.96, 128.93, 128.87, 126.90, 123.45, 101.84, 54.20, 47.66; Elemental analysis: Calc: C, 77.63; H, 4.74; N, 8.23. Found: C, 77.89; H, 4.52; N, 8.45. (Mol. Formula: C_22_H_16_N_2_O_2_.

### 3.3. Synthesis of [Ru(II)-L_1_L_2_(NCS)_2_

[RuCl_2_(dmso)_4_], used as metal precursor, was synthesized as reported [26]. The [RuL_1_L_2_(NCS)_2_] complex was synthesized following the literature procedure [27] with slight modifications as follows: In a 250 mL flask, [RuCl_2_(dmso)_4_] (0.095 g, 0.20 mmol) was dissolved in *N,N*-dimethylformamide (40 mL) followed by successive addition of L_1_ (0.41 g, 0.59 mmol) and L_2_ (0.20 g, 0.59 mmol) and excess aqueous solution of NH_4_NCS (0.90 g, 0.01 mmol, 10% excess). The mixture was refluxed in the dark under argon at 150 ºC for 8 h. After the reaction, the solution was allowed to cool to room temperature and filtered to remove unreacted starting materials. The filtrate was concentrated to dryness and 0.05 M NaOH solution (40 mL) was added to give a dirty brown precipitate which was filtered off. The pH of the resulting solution was adjusted to 3 with 0.5 M HNO_3_. The solution was left to stand in the fridge (-2 ºC) for 12 h before filtration and concentration *in vacuo*. Water was added to the semisolid to remove excess NH_4_NCS. The water insoluble product was collected on sintered glass crucible by suction filtration and washed with diethyl ether and dried in the oven at a moderate temperature. The resulting crude complex was dissolved in methanol and carefully adsorbed on the Sephadex LH 20 column chromatography using chloroform-methanol (1:1) as mobile phase. A dark-brown solid product was isolated after evaporation of solvent and subsequent recrystallization in ethanol-diethyl ether mixture. Yield 0.45 g, 28.4%; m.p. 252-254 ºC, R_f_ = 0.63, solvent system D: chloroform-methanol (1:1); FT-IR: (KBr pellet) 3,550, 3,472, 3,14, 3,236 (OH, α,β- unsaturated carboxylic acid); 3,052 (=C-H_str_), 2,925, 2,853 (CH_3_), 2,107 (br, N=C_str,_ C≡C), 1,972, 1,637 (C=O_str_); 1,618 (C=C_str_), 1,543 (C=N_str_); 1,496, 1,435 (C=C_str_ *alkene*); 1,385 (C=O_sym_), 1,304, 1,256, 1,162, 1,119, 1,094 (C-H), 1,028 (C-O_str_), 998 (C-H), 926, 843, 800, 748 (C-H_arom_), 721, 697, 619, 528, 473 (Ru-N); ^1^H NMR (DMSO-d_6_) δ 8.53 (d, *J* = 2.8 Hz, 1H), 8.52 (d, *J* = 2.8 Hz, 1H), 8.17 (d, *J* = 2.2 Hz, 4H), 7.79 (dd, *J* = 2.8, 6.8 Hz, 8H), 7.62 (d, *J* = 4.0 Hz, 2H), 7.58 (d, *J* = 11.2 Hz, 2H), 7.56 (d, *J* = 8.0 Hz, 2H), 2.08 (s, CH_3_); UV: (ε, M^‑1^cm^-1^): 343 (50,490), 363 (69,570), 384 (98,155), 405 (100,000), 461 (41,400), 915 (10,570), 1008 (9,080); Elemental Analysis: Calc.: C, 72.26; H, 4.13; N, 5.75, S, 4.38 Found: C, 72.52; H, 4.46; N, 5.63. Molecular Formula: [C_88_H_60_N_6_O_6_S_2_Ru].

## 4. Conclusions

A heteroleptic [RuL_1_L_2_(NCS)_2_] complex containing two different types of functionalized anthracenyl derivatives as well as a 1,3-enyne molecular system on phenanthroline ligands was synthesized and characterized. A preliminary investigation of the photophysical properties of the [RuL_1_L_2_(NCS)_2_] complex showed interesting absorption and high luminescent intensity covering a wide range in the visible region of the spectrum. The improved photophysical properties were ascribed to the incorporation of a long chain π-bond conjugated coupling of the mono- and dianthracene linkage as well as the double-triple π-bond linkage of the 1-methoxy-1-buten-3-yne functionalities on the phenanthroline ligands. The [RuL_1_L_2_(NCS)_2_] complex may be a suitable candidate for larger supramolecular systems based on Ru(II) polypyridine compounds capable of performing long-range photoinduced electron and/ or energy transfer functions. To support the potential capability of this complex as a sensitizer for dye-sensitized solar cells, the electrochemical and photovoltaic performance experiments are in progress.

## Supplementary Materials

Supplementary materials can be seen at http://www.mdpi.com/1420-3049/15/11/7570/s1.
