# Supplementary material for: Synthesis and Characterization of a Ru(II) Complex with Functionalized Phenanthroline Ligands Having Single-Double Linked Anthracenyl and 1-Methoxy-1-buten-3-yne Moieties
Source: Molecules. 2010 Oct 27;15(11):7570–81. doi: 10.3390/molecules15117570 (PMC6259579; doi:10.3390/molecules15117570)

Supplementary Materials

S1: IR spectra of L_1: 4-(9-dianthracenyl-10-(2,3-dimethylacrylic acid)-7-(9-anthracenyl-10-(2,3-dimethylacrylic acid)-1,10-phenanthroline (top) and 9,10-dibromoanthracene (bottom).

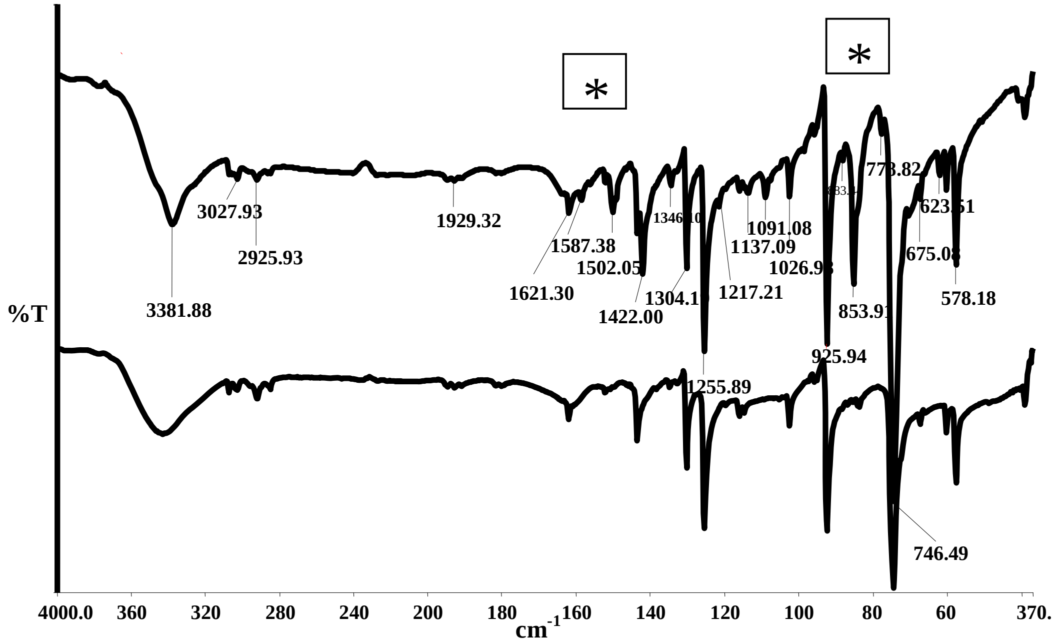

S2: IR spectra of L_2: 4,7-Bis(1-methoxy-1-buten-3-yne)-phenanthroline.

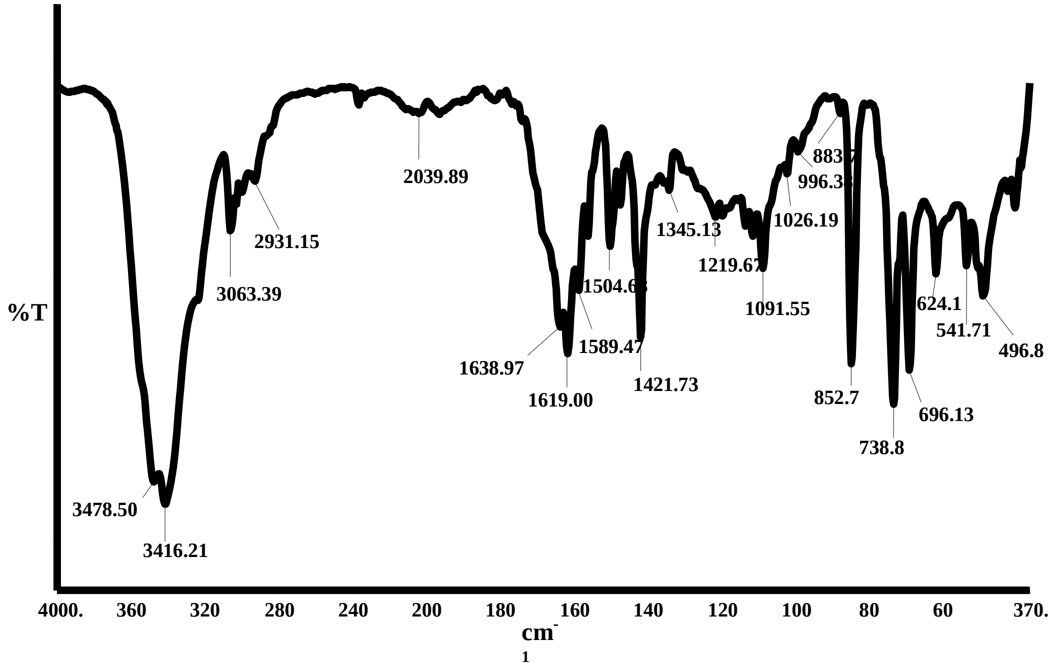

S3: IR spectra of Ru(II)$L_1L_2(NCS)_2$ complex.

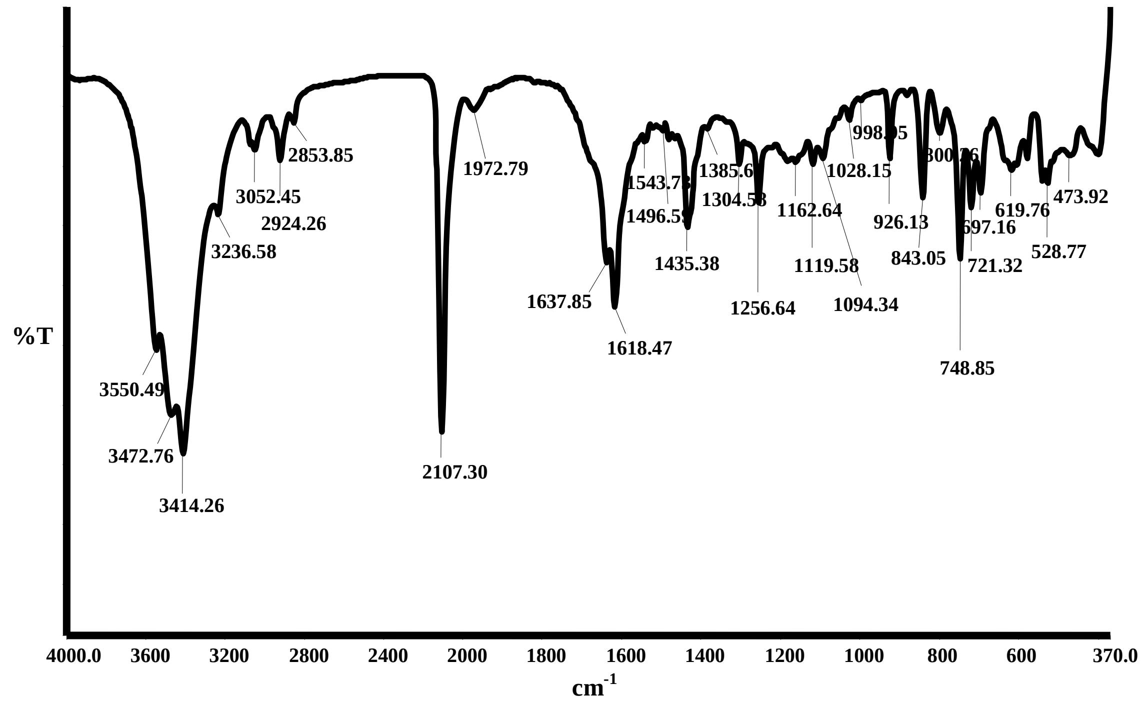

S4: ^{1}H NMR spectra of $L_{1.}$

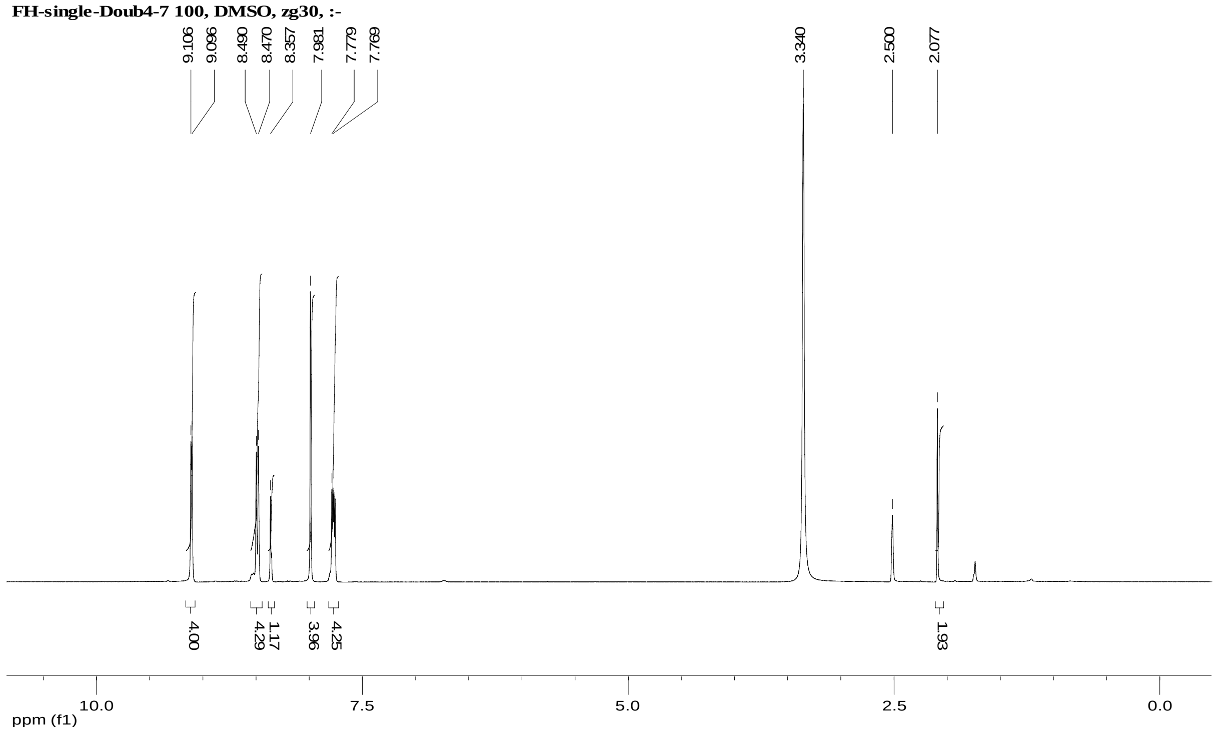

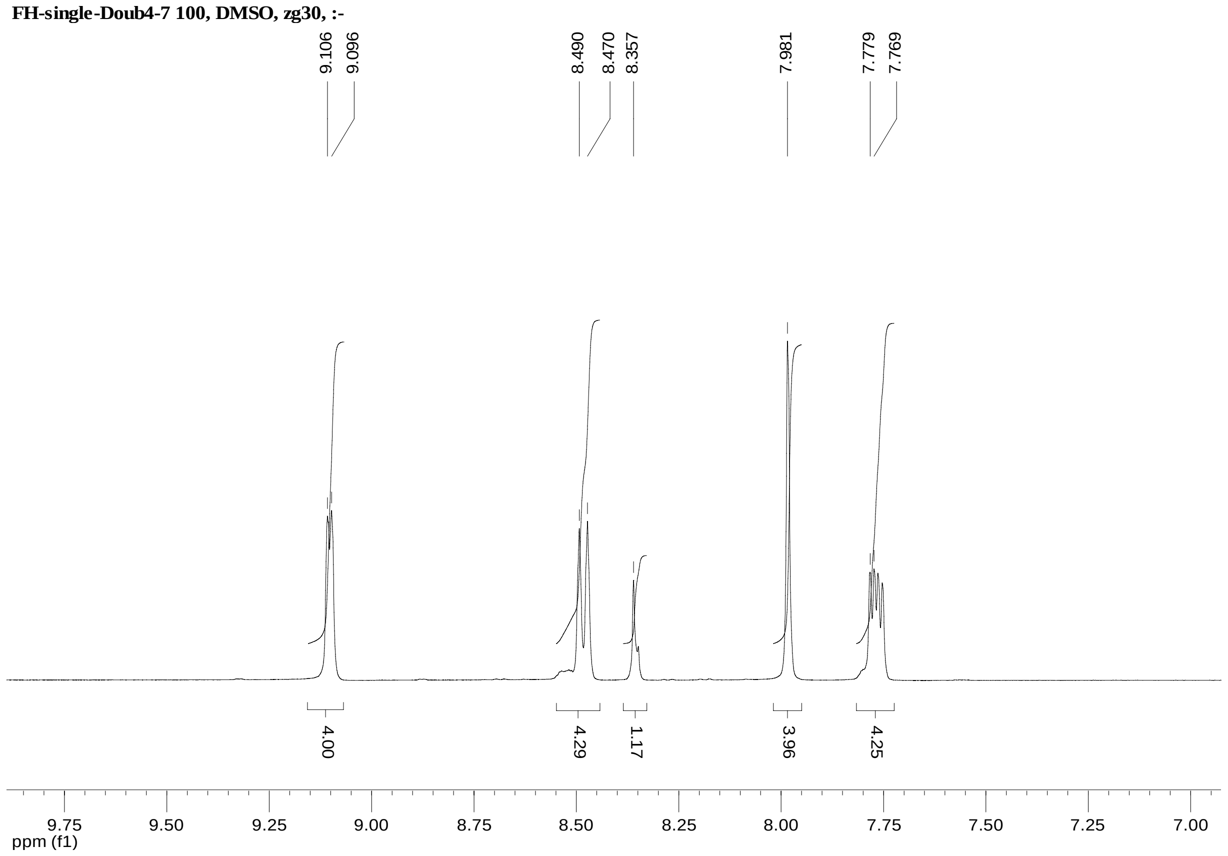

S5: ^{13}C NMR spectra of $L_{1.}$

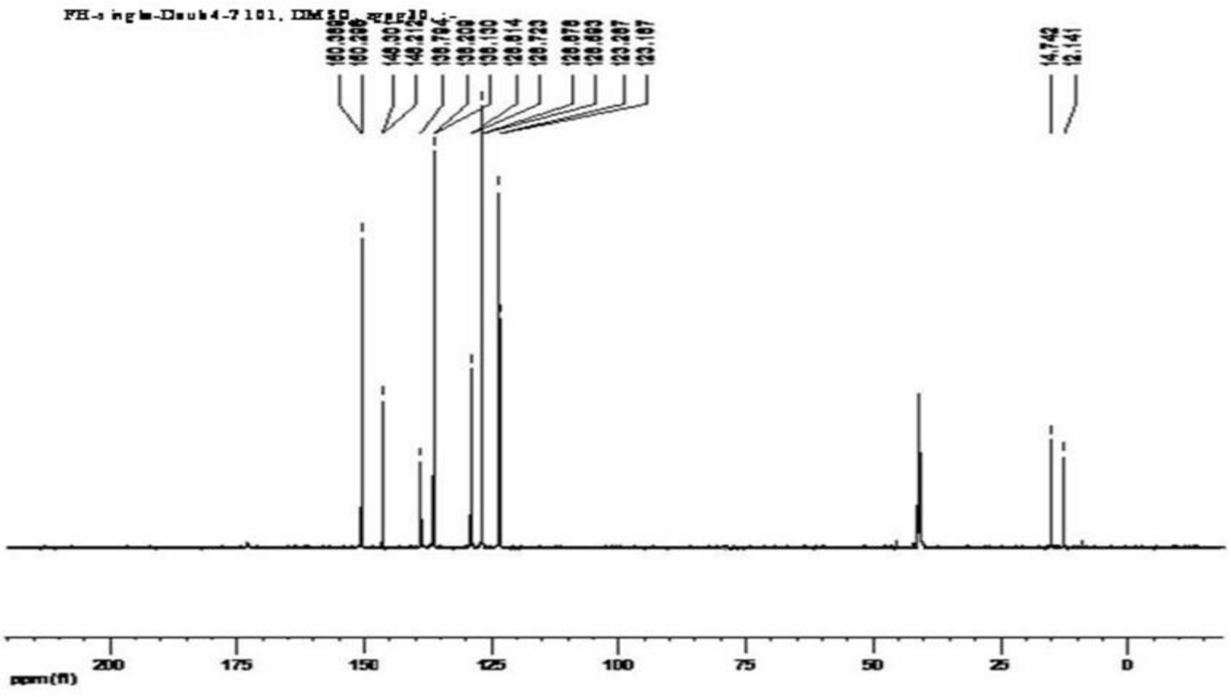

S6: ^{1}H NMR spectra of $L_{2.}$

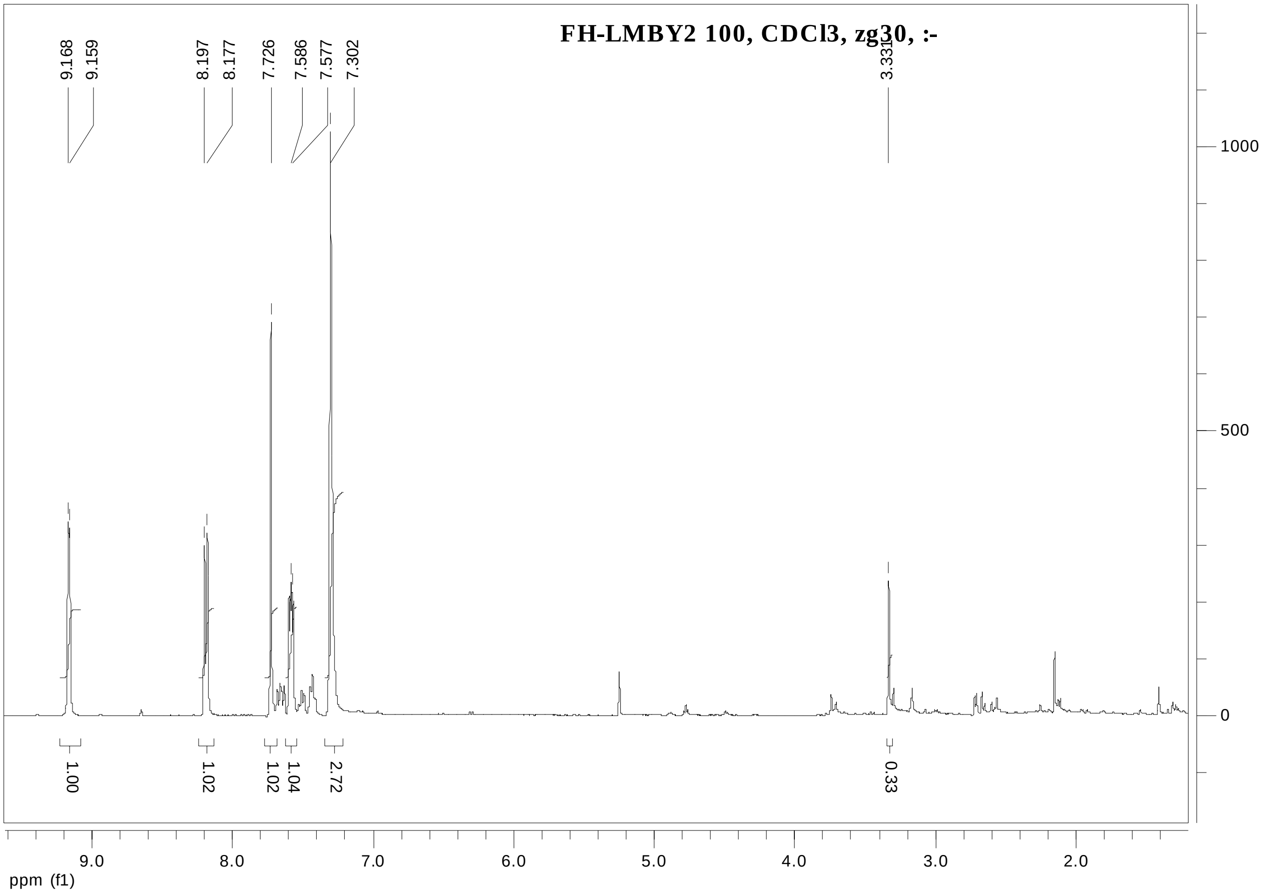

S7: ^{13}C NMR spectra of $L_{2.}$

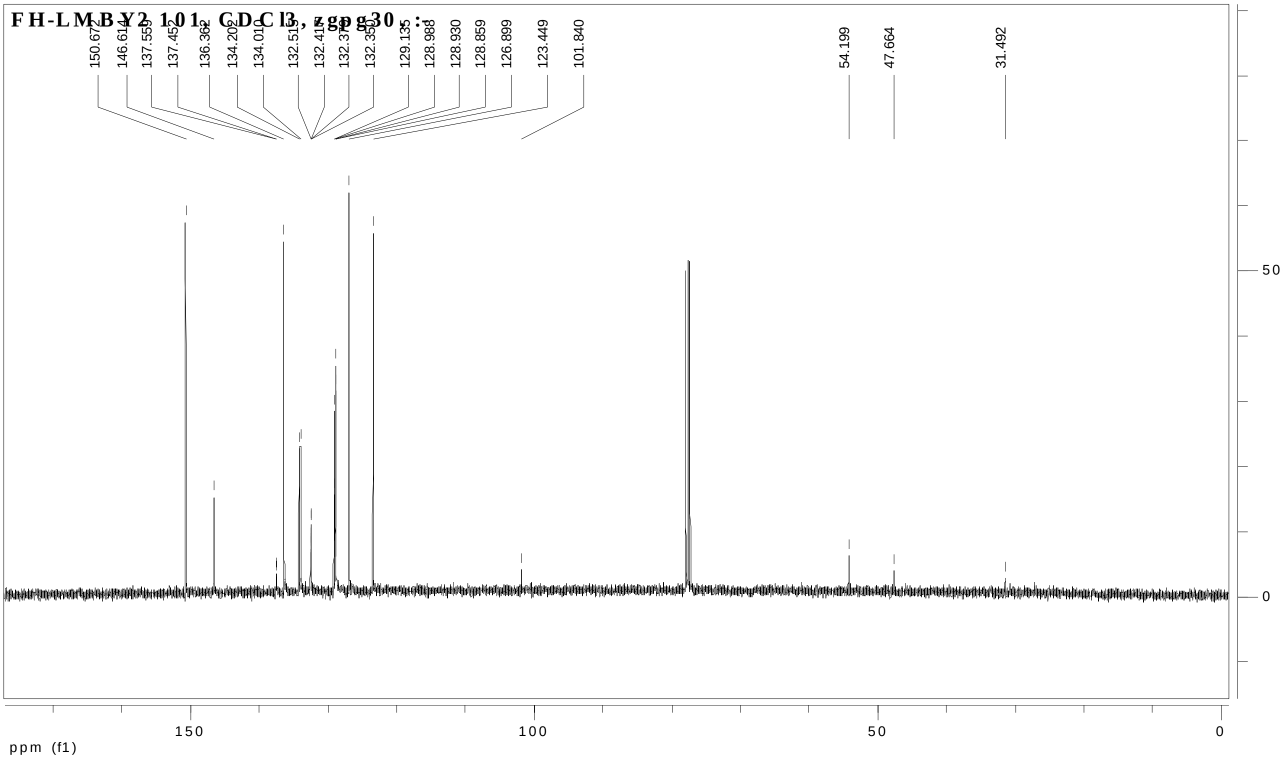

Supplement: Supplementary file 1 [file molecules-15-07570-s001.pdf]
